# *In vivo* tunable CRISPR mediates efficient somatic mutagenesis to generate tumor models

**DOI:** 10.1007/s13238-018-0579-7

**Published:** 2018-09-29

**Authors:** Xiaomeng An, Linlin Li, Sen Wu

**Affiliations:** 1 State Key Laboratory of Agrobiotechnology, College of Biological Sciences, China Agricultural University, Beijing, 100193 China; 2 State Key Laboratory of Veterinary Etiological Biology, National Foot and Mouth Diseases Reference Laboratory, Key Laboratory of Animal Virology of Ministry of Agriculture, Lanzhou Veterinary Research Institute, Chinese Academy of Agricultural Sciences, Lanzhou, 730000 China

**Dear Editor,**

CRISPR/Cas9 has revolutionized genome editing technology due to its simplicity and robustness (Mali et al., 2013). Several inducible CRISPR/Cas9 systems recently developed make spatiotemporal genome editing possible (Konermann et al., 2013; Balboa et al., 2015; Dow et al., 2015; Zetsche et al., 2015; Liu et al., 2016; Kleinjan et al., 2017; Maji et al., 2017; Senturk et al., 2017; Lu et al., 2018). However, whether these inducible CRISPR/Cas9 systems can mediate efficient *in vivo* somatic mutagenesis for tumor modeling remains to be tested. Destabilizing domain (DD), a member of the post-translationally inducible elements, can be fused to Cas9 protein to construct an inducible CRISPR/Cas9 system (Senturk et al., 2017). The DD tag guides newly synthesized DD-Cas9 protein to the proteasome for degradation, which can be abrogated by ligand trimethoprim (TMP) (Fig. 1A) (Iwamoto et al., 2010; Sando et al., 2013). Because of the convenience of TMP delivery and no endogenous targets found for TMP in mammals, TMP-inducible Cas9 stabilization would be extremely attractive for achieving post-translationally tunable genome editing *in vivo*.

**Figure 1:**
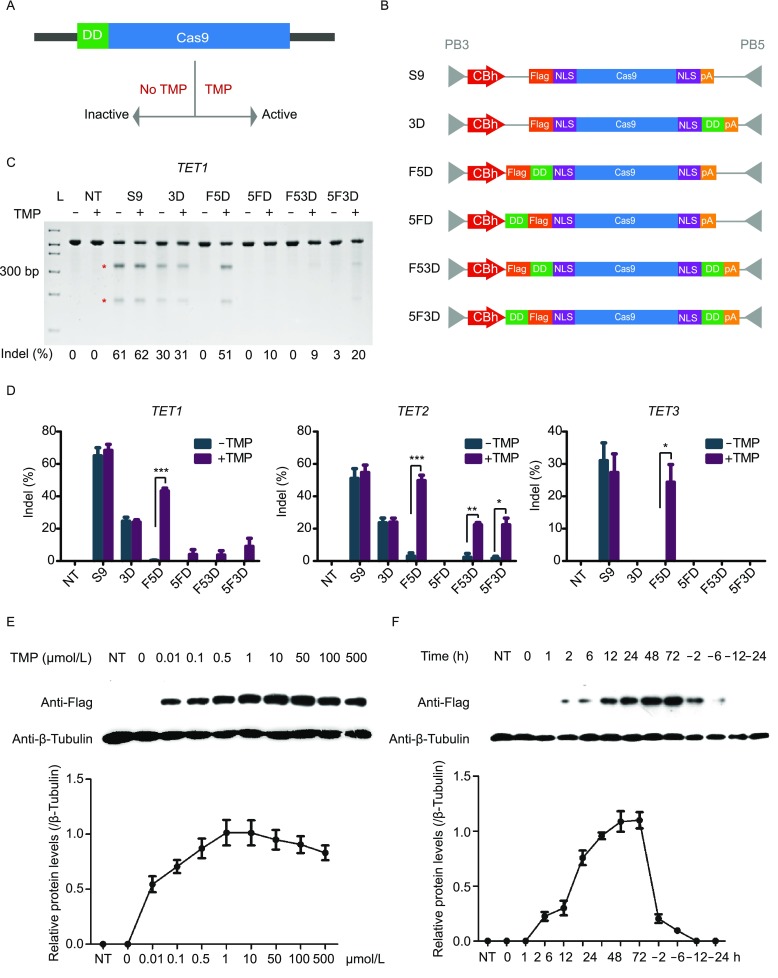
**TMP-inducible DD-Cas9 constructs**. (A) Schematic of the DD-Cas9 strategy. DD-Cas9 is inactive (degraded) in the absence of a cell-permeable small molecule trimethoprim (TMP), and is active (fully functional) in the presence of TMP. (B) DD-Cas9 variants. Top row, the constitutive wild-type Cas9 expression plasmid S9, which contains a CBh promoter, a Flag tag, two NLSs joined to Cas9 enzyme. Other rows represent five DD-Cas9 variants with distinct configurations of Flag tag, DD, and Cas9 with their abbreviations on the left. PB3 and PB5, terminals of the *piggyBac* transposon; pA, polyadenylation signal. (C) Representative T7EI cleavage assay of DD-Cas9 variants at *TET1* locus. In HeLa cells, the editing activity of these DD-Cas9 variants was evaluated with and without 1 μmol/L TMP treatment for 5 days. Red asterisks indicate the expected T7EI-specific cleavage bands. Relative indel frequencies (%) were illustrated at the bottom line. L, 1 kb plus DNA ladder; NT, non-transfected group. (D) Bar charts illustrated the targeting efficiencies of *TET1*, *TET2*, *TET3* by T7E1 assay in the absence or presence of TMP. F5D was tightly controlled by TMP and displayed significantly higher activity across all three targeted genes. Data indicate the means ± SD of three biological replicates (*P < 0.05, **P < 0.01, ***P < 0.001, Student’s *t*-test). (E) Determination of the optimal TMP working concentration of F5D. HeLa cells expressing F5D were exposed to TMP with increasing concentrations for 5 days. The Cas9 protein levels were analyzed by Western blot with an anti-Flag antibody (top). β-Tubulin was used as a loading control. The corresponding protein levels were quantified using the ImageJ software, and the indicated ratios were graphed (bottom). The results show that Cas9 protein level reaches a plateau at 1 μmol/L TMP. Data indicate the means ± SEM of three independent experiments. (F) Kinetics of DD mediated Cas9 protein degradation. Western blot analysis was performed for DD-Cas9 proteins obtained from continuous TMP (1 μmol/L) exposure (up to 72 h) or withdrawal after 2 h TMP treatment at indicated time points (top). The indicated ratios were graphed (bottom). Data indicate the means ± SEM of three independent experiments.

To obtain an efficient and tightly controlled DD-Cas9, we created five variants with distinct configurations, all on a *piggyBac* transposon plasmid backbone (Fig. 1B). We next determined the activities of these Cas9 variants by targeting three human genes (*TET1*, *TET2* and *TET3*) by T7 Endonuclease I (T7EI) assay (Figs. 1C, 1D and S1). In the presence or absence of TMP, no insertion/deletion (indel) was detected in the non-transfected (NT) control group (Fig. 1D). Among these five variants, F5D (DD fused to N-terminal of Cas9 protein) displayed significantly higher activity than other variants when TMP was added, but demonstrated no activity in the absence of TMP (Fig. 1D). Hence, the F5D construct was used in all subsequent experiments. To optimize the working concentration of TMP, we examined Cas9 protein expression from F5D under eight TMP concentrations. At 0.01 μmol/L TMP, stabilized Cas9 protein started to show up. As TMP concentration increased, Cas9 protein level increased gradually as well and reached a plateau at 1 μmol/L TMP (Fig. 1E). Therefore, 1 μmol/L TMP was used in all subsequent *in vitro* studies. Finally, we assayed the kinetics of DD mediated protein degradation. Cells were treated with TMP for 1, 2, 6, 12, 24, 48, 72 h and harvested for Cas9 detection by Western blot. As soon as 2 h after TMP treatment, rapid Cas9 stabilization was detected and became undetectable 12 h after TMP withdrawal (Fig. 1F). In summary, these results demonstrated that F5D is highly efficient for *in vitro* genome editing and the DD-Cas9 is tightly controlled by TMP at the protein level.

Our previous work demonstrated that tail vein injection of sgRNAs for *Cdkn2a* and *Trp53*, an hNRAS^G12V^ overexpression plasmid, along with Cas9 expression plasmid achieved almost 100% liver tumorigenesis in mice (Xu et al., 2017). To determine whether DD-Cas9 works *in vivo* in a tunable manner, 26 mice were randomly separated into four groups and injected through tail vein with different plasmid combination (Table 1 and Fig. S2) to induce tumors (Fig. 2A). When examined six weeks after injection, mice in group A (no F5D, no TMP), group B (no F5D, +TMP) and group C (F5D, no TMP) were all tumor-free (Fig. 2B). In group D (F5D, +TMP), liver tumors of various sizes with strong GFP fluorescence were found in eight of ten injected mice (Fig. 2B). These data suggested that F5D could induce tumors in a tunable manner as anticipated. Next, we performed T7EI assay and confirmed the successful gene targeting of both *Cdkn2a* and *Trp53* in tumor tissues (Figs. 2C and S2B). In addition, TA-cloning and Sanger sequencing confirmed indel mutations at the two genomic loci (Fig. 2D). Finally, we carried out histological analysis and proliferation detection via H&E and Ki67 staining respectively. Compared with normal liver sections, tumors induced showed a tubular growth morphology and were highly proliferative (Fig. 2E). Taken together, these results indicated that DD-Cas9 can inducibly mediate efficient liver tumorigenesis in adult mice, and DD-Cas9 can be exploited *in vivo* to engineer endogenous genes in an inducible manner.

**Table 1:** DD-Cas9 can drive tumorigenesis in an inducible manner in mouse livers.

Mouse group	F5D	Cdkn2a-sgRNA-hNRAS^G12V^	Trp53-sgRNA	CAG-PBase	TMP supplied	Tumorigenesis efficiency (%)
A	0 μg	12 μg	12 μg	8 μg	No	0/4 (male, 0%)
B	0 μg	12 μg	12 μg	8 μg	0.5 mg/mL	0/4 (male, 0%)
C	8 μg	8 μg	8 μg	8 μg	No	0/8 (male, 0%)
D	8 μg	8 μg	8 μg	8 μg	0.5 mg/mL	8/10 (male, 80%)

**Figure 2:**
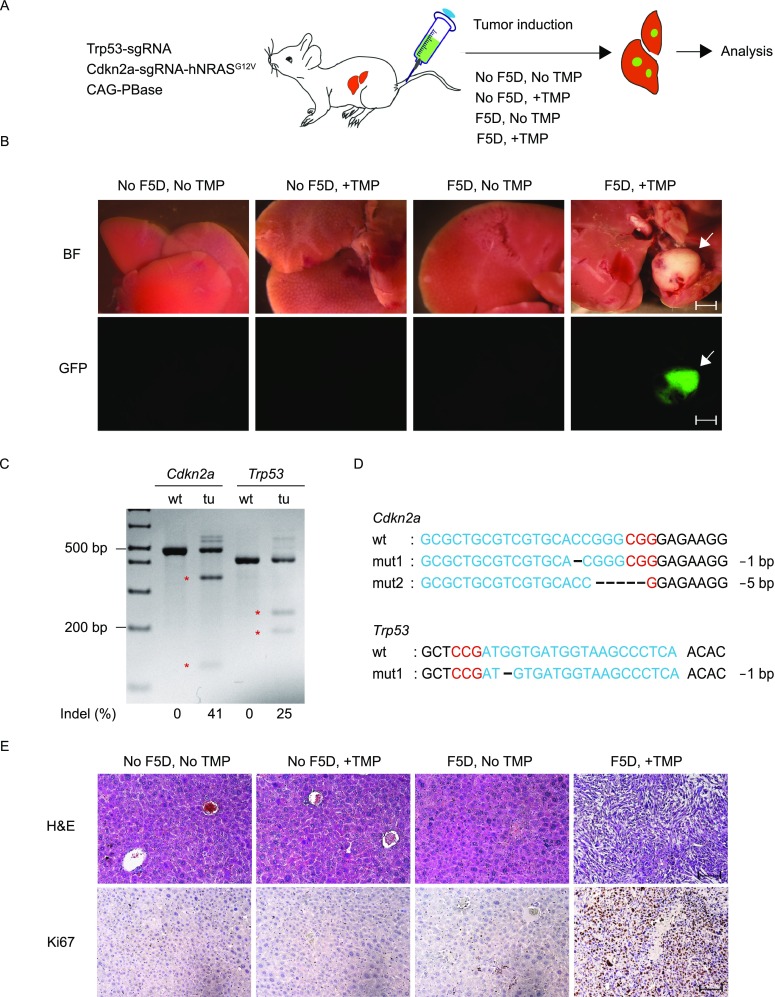
**DD-Cas9 mediated liver tumorigenesis in an inducible manner**. (A) Workflow of the induction of liver tumors in mice by hydrodynamic tail vein injection. (B) Representative liver tumors obtained from the mice under different treatments. Mice injected F5D and induced with TMP developed tumors with GFP fluorescence. White arrows indicate tumor sites. Scale bar, 100 μm. (C) T7EI cleavage assay of genome modification in a representative liver tumor. Red asterisks indicate the expected T7EI-specific cleavage bands used to quantify indel frequencies (the bottom line). Tu, tumor sample. (D) Sequences of indel mutation in a representative liver tumor. Protospacer-adjacent motif (PAM) sequences were labeled in red and sgRNA coding sequences were labeled in cyan. (E) H&E and immunohistochemistry analysis of the indicated liver samples. Slides showed that tumor cells grew in a tubular pattern and proliferated rapidly by highly expressing Ki67 in contrast to the other liver tissues. Scale bar, 100 μm.

TMP is inexpensive, non-toxic and can penetrate the placental barrier and the blood-brain barrier. All these advantages make TMP ideal to stabilize DD tagged proteins *in vivo* (Iwamoto et al., 2010). In the current study, we fused DD to different domains of Cas9 to form five variants with the purpose of tuning Cas9 expression at the protein level. Although F5D met our demands well both *in vitro* and *in vivo*, more configurations could be tested for other purposes. In conclusion, our successful *in vivo* delivery of an inducible CRISPR-Cas9 may provide a foundation for future safer gene therapy.

## Electronic supplementary material

Below is the link to the electronic supplementary material.

Supplementary material 1 (PDF 6047 kb)
