# Supplementary material for: In vivo tunable CRISPR mediates efficient somatic mutagenesis to generate tumor models
Source: Protein Cell. 2018 Sep 29;10(6):450–4. doi: 10.1007/s13238-018-0579-7 (PMC6538589; doi:10.1007/s13238-018-0579-7)

Supplemental materials

Materials and Methods

Plasmids.

To construct the plasmid S9, SV40-neo from pIRES2-EGFP (Clontech), the PB 5′ and 3′ terminals from the ZGs plasmid were amplified and inserted into plasmid pX330 (pX330-U6-Chimeric_BB-CBh-hSpCas9), which is from Addgene depository of Feng Zhang lab (Addgene plasmid #42230). Plasmid S9 contains a human codon-optimized *S. pyogenes* Cas9 enzyme driven by CBh promoter, a Flag tag at the N-terminus of Cas9 enzyme and one copy NLS at the N and C terminus of Cas9 enzyme respectively. Plasmid S9 was used as the constitutive wild-type Cas9 expression plasmid. Then we synthesized a human codon-optimized gene that codes for high-affinity ligand dependent destabilization domain (DD) derived from Escherichia coli dihydrofolate reductase (ecDHFR), with the R12Y and Y100I mutations. All of the DD-Cas9 variants plasmids were constructed based on plasmid S9 using the Gibson Assembly method (New England Biolabs). Plasmid S9 was digested with restriction enzyme AgeI/FseI and gel purified as the backbone using the QIAquick gel extraction kit (Qiagen). Each of the DD fragment flanked with AgeI or FseI sites were cloned into the N or C terminus of the Cas9 enzyme respectively. S9 with only one copy DD added to the C terminus was named 3D; one copy DD added to the N terminus of Cas9 enzyme upstream or downstream of the Flag-tag, referred to as 5FD and F5D respectively; besides, F53D, 5F3D both contain two copy DD at the N and C terminus. These five DD-Cas9 variants were all delivered by *piggyBac* transposon with PB

transposase (plasmid pCAG-PBase) used together. All Cas9 variants were confirmed by Sanger sequencing. Plasmid DNA for tail vein injection (pPB-Cdkn2a-sgRNA-hNRAS^{G12V}-IRES-eGFP, Trp53-sgRNA, pCAG-PBase and F5D) were prepared by Qiagen EndoFree Plasmid Maxi Kit. Various sgRNAs were designed by online CRISPR design tool (http://tools.genome-engineering.org). Subsequently, oligos (see primer sequence in Table S1) were annealed and cloned into corresponding vector backbone digested by BbsI.

Cell culture and transfection.

HeLa cells (CLS, Cell Lines Service) were cultured in Dulbecco′s modified Eagle medium (DMEM, Invitrogen) supplemented with 10% (vol/vol) fetal bovine serum (FBS, Gibco), 1% penicillin–streptomycin (Gibco), 1 mM Sodium Pyruvate (Invitrogen) and 1% non-essential amino acids (Invitrogen). The cells were dissociated by 0.1% Trypsin (Invitrogen) and expanded when cells grew around 70% confluence. The cells were incubated in a humidified 37 ℃, 5% CO_2 incubator with culture media replaced every day. TMP (Sigma), solubilized in DMSO (Sigma), was added to culture media with given concentrations.

One million cells were electroporated with 3 μg DD-Cas9-sgRNA variants and 1 μg CAG-PBase with Nucleofector™ 2b Device (Lonza) with program I-013 according to the manufacturer′s instructions. One day after electroporation, HeLa cells were selected in DMEM containing 1 mg/mL G418 (Gibco) for 5 days. Subsequently, 1 μM TMP was added to culture media of the experimental group cells,

whereas control cells were kept in culture medium without TMP for 5 days. Genomic DNA was extracted for subsequent experiments.

T7EI cleavage assay

The genomic DNA was extracted under manufacturer′s instructions (Invitrogen). After an initial treatment in a thermal cycler (99 ℃ for 10 minutes), genomic regions flanking the CRISPR target sites were PCR amplified using Herculase II Fusion Polymerase (Stratagene) (see primer sequence in Table S1). The PCR products were purified using the QIAquick gel extraction kit (Qiagen). Purified PCR fragments (~400 ng) mixed with 2 μL 10 × Taq DNA Polymerase PCR buffer in a total volume of 20 μL were subjected to a series of melting and reannealing temperature cycles with gradual decrements in each thermal cycle. After annealing, 5 U T7 endonuclease I (New England BioLabs) was added to each sample and the reactions were incubated at 37 ℃ for 60 min. Subsequently, the digested fragments were resolved on the polyacrylamide gel and then quantified using ImageJ software. Indel percentage was calculated by the formula $100 \times (1- (1- (b + c) / (a + b + c))^{1/2})$. In this formula, "b" and "c" represent the gray value of the cleaved bands while "a" represents the gray value of the un-cleaved band.

Mice and tail vein injection.

All institutional and national guidelines for the care and use of laboratory animals were followed.

In this study, all the mouse experiments were approved by the institutional animal care and use committees at China Agricultural University. For hydrodynamic liver

injection, all plasmids, suspended in 3mL saline (~10% of body weight), were injected via the tail vein in 5-7 seconds into 4 weeks old CD-1 male mice purchased from Charles River. Mice were randomly distributed into four groups. Detail information about plasmid combination during injection and tumorigenesis efficiency were illustrated in Table 1. 8 mice in group C were kept off TMP water whereas 10 mice in group D were treated with TMP (0.5 mg/mL, 1.72 mM) in water continuously throughout the experiment. Until six weeks post-injection, all mice were examined. Liver tumor tissues were collected for genomic DNA extraction and fixed in freshly prepared 4% paraformaldehyde for 24 h.

Western blot.

Protein samples were isolated by resuspending cell pellets in cell lysis buffer for Western blot (20 mM Tris at pH 7.5, 150 mM NaCl, 1% Triton X-100, sodium pyrophosphate, β-glycerophosphate, EDTA, leupeptin; Beyotime). After removal of the debris, samples were quantified with BCA Protein Assay Kit (Beyotime). After being denatured at 99 ℃ for 10 min, 20 μg total proteins were electrophoresed on 10% gradient gels and wet-transferred to Immobilon-P transfer membrane (Millipore). After being blocked with 5% nonfat dry milk in 1×TBS, 0.1% Tween20 at room temperature for 1 h, membranes were incubated with specific primary antibodies such as Flag mouse monoclonal (1:1,000, Beyotime) or β-Tubulin mouse monoclonal (1:2,000, Easybio) at 4 ℃ overnight. Subsequently, membranes were rinsed five times with 1×TBS-T and then incubated with HRP labeled goat-anti-mouse IgG secondary antibody (1:5,000, Beyotime) at room temperature for 2 h. Then the membranes were

washed again and incubated with SuperSignal West Dura Extended Duration Substrate (Thermo) according to the manufacturer′s instructions. Finally, the membranes were exposed to X-ray film (Fuji film).

Histology and immunohistochemistry.

Tumor samples were fixed in 4% (wt/vol) paraformaldehyde at 4 ℃ for 24 h, paraffin embedded, cut in 5 μm sections and stained with hematoxylin and eosin (H&E) for histopathological analysis. For immunohistochemistry, sections were deparaffinized, hydrated, heated to expose antigenic, treated with 1% H_2O_2 to remove endogenous peroxidase and 0.2% Triton X-100, then blocked at room temperature for 1 h. Incubation with primary antibody Ki-67 (Thermo Scientific, RM-9106, 1:200) was performed overnight at 4 ℃. Subsequently, streptavidin peroxidase staining was performed following the description of the manufacturer (Zsbio).

Supplementary Figure Legends

Figure S1. Schematic of genomic locations of three human gene and targeting efficiencies.

(A) Human gene loci *TET1*, *TET2,* and *TET3.* Grey rectangular and white number denote corresponding exon location. Magenta arrows illustrate the location of sgRNAs. Dark blue arrows stand for forward and reverse primers used to amplify fragments around sgRNA target sites. Protospacer-adjacent motif (PAM) sequence was labeled in magenta, and sgRNA coding sequences were labeled in black.

(B) T7EI cleavage assay of DD-Cas9 variants at *TET1* locus. ex1, ex2 and ex3 denote

three biological replicates. "-" and "+” denote the absence or presence of 1 μM TMP, respectively.

(C) T7EI cleavage assay of DD-Cas9 variants at *TET2* locus.

(D) T7EI cleavage assay of DD-Cas9 variants at *TET3* locus.

Figure S2. Plasmids used in tail vein injection in mice and mouse gene loci of *Cdkn2a* and *Trp53*.

(A) Plasmids used in tail vein injection in mice and their abbreviations. U6, human U6 promoter; PB3 and PB5, terminals of the *piggyBac* transposon; hNRAS^{G12V}, amplified from human cDNA.

(B) Mouse gene loci *Cdkn2a* and *Trp53.*

Supplementary Table

Table S1 list of primers

Primer Name	Primer sequence
**sgRNA sequence**	
hTET1sgRNA-S	CACCGGCCCATATTATACACACCT
hTET1sgRNA-A	AAACAGGTGTGTATAATATGGGCC
hTET2sgRNA-S	CACCGCTATCAAGTTCTGCAGCAG

hTET2sgRNA-A	AAACCTGCTGCAGAACTTGATAGC
hTET3sgRNA-S	CACCGATCGAGAAGGTCATCTACA
hTET3sgRNA-A	AAACTGTAGATGACCTTCTCGATC
mCdkn2a-sgRNA-S	CACCGCGCTGCGTCGTGCACCGGG
mCdkn2a-sgRNA-A	AAACCCCGGTGCACGACGCAGCGC
mTrp53-sgRNA-S	CACCGTGAGGGCTTACCATCACCAT
mTrp53-sgRNA-A	AAACATGGTGATGGTAAGCCCTCAC

PCR primers used for Surveyor assay

hTET1-F	GGAGATAGGAGTATAAATATGACCC
hTET1-R	GCCCTAAGAAACATCCAACTC
hTET2-F	ACACAGCAACCCCAAACTG
hTET2-R	TTGCTAATTCTGGATAAACGC
hTET3-F	CCCTTCTGGAGTTCGGAG
hTET3-R	ACAGTTCCAAGGCTGACCC
mCdkn2a-F	CAGCGGGTGGGTAAAATGGGAACT
mCdkn2a-R	ACTCTGGCCGTGATCCCTCTACTT

mTrp53-F	CCCCACCTTGACACCTGATCGTTA
mTrp53-R	CCTCGGGTGGCTCATAAGGTACCA

Vector construction primers

AXM-118-5DD-S9-F	AAATCACTTTTTTTCAGGTTGGACCGGT GCCACCATGATCAGCCTGATCGCCGC
AXM-118-5DD-S9-R	TCCGTCGTGGTCCTTATAGTCCATG CGGCGCTCCAGGATCTCGAAGCAG
AXM-119-FLAG-5DD-F1	GAAATCACTTTTTTTCAGGTTGGACCGGT GCCACCATGGACTATAAGGACCACGAC
AXM-119-FLAG-5DD-R1	GCGGCGATCAGGCTGATCATCTTATC GTCATCGTCTTTGTAATC
AXM-119-FLAG-5DD-F2	GATTACAAAGACGATGACGATAAGA TGATCAGCCTGATCGCCGCCCTG
AXM-119-FLAG-5DD-R2	CTTCCGCTTCTTCTTTGGGGCCATGC GGCGCTCCAGGATCTCGAAGCAG
lin565-3DD-F	AAAAGGCCGGCGGCCACGAAAAAG GCCGGCCAGGCAAAAAAGAAAAAG

lin566-3DD-R	ATGATCAGCCTGATCGCCGCC<br>GAGCTCTAGGAATTCTTAGCGG<br>CGCTCCAGGATCTCGAA
lin567-DD seq-F	GTACGGTGGGCGCCTATAAAA
lin568-DD seq-R	CTTGTACTCGTCGGTGATCAC
AXM-T-1-3'DD-F	GAGCATCACCGGCCTGTACGAGAC
AXM-T-1-3'DD-R	AGTGGGAGTGGCACCTTCCAG

Supplementary Figure 1

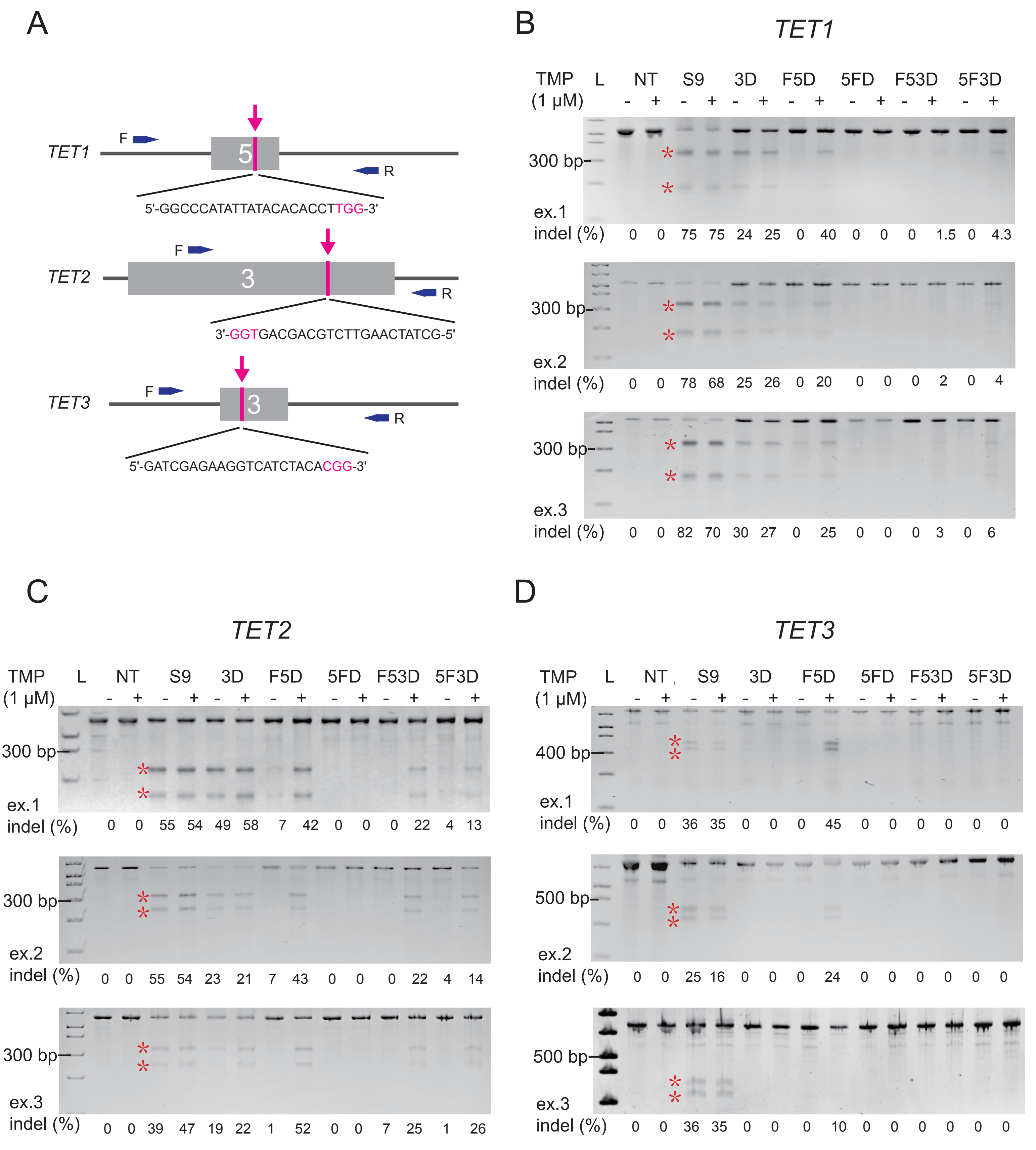

Supplementary Figure 2

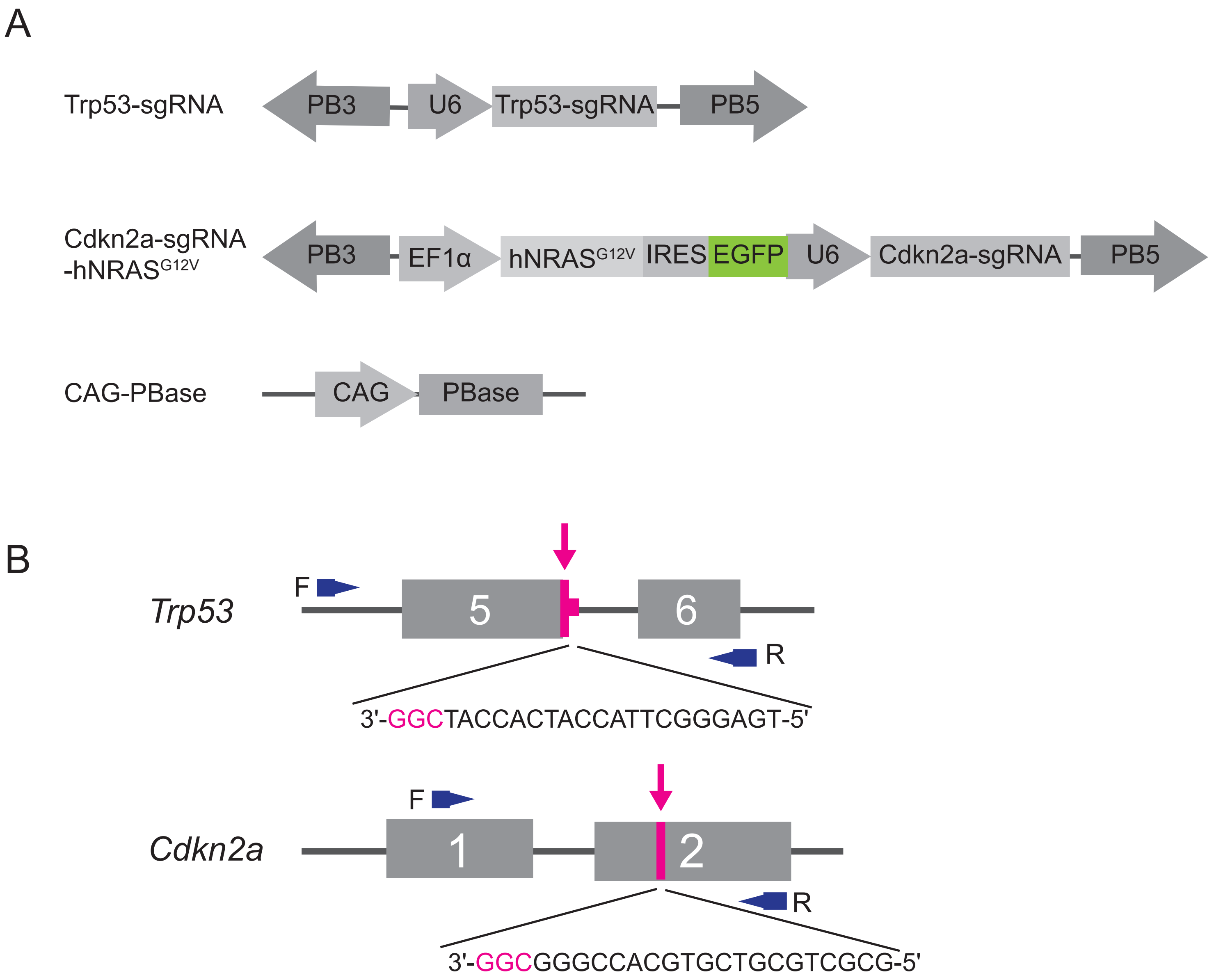

Supplement: Supplementary file 1 — Supplementary material 1 (PDF 6047 kb) [file 13238_2018_579_MOESM1_ESM.pdf]
